# Exploring drivers and barriers to the utilization of community client-led ART delivery model in South-Western Uganda: patients’ and health workers’ experiences

**DOI:** 10.1186/s12913-021-07105-9

**Published:** 2021-10-20

**Authors:** Timothy Mwanje Kintu, Anna Maria Ssewanyana, Tonny Kyagambiddwa, Pretty Mariam Nampijja, Patience Kevin Apio, Jessica Kitaka, Jerome Kahuma Kabakyenga

**Affiliations:** Faculty of Medicine, Mbarara University of Science and Technology, P.O. Box 1410, Mbarara, Uganda

**Keywords:** CCLAD, Utilization, HIV, Uganda, Differentiated service delivery, Client-led, ART delivery

## Abstract

**Background:**

In an effort to accommodate the growing number of HIV clients, improve retention in care and reduce health care burden, the differentiated service delivery (DSD) models were introduced in 2014. One such model, Community Client-Led ART Delivery (CCLAD) was rolled out in Uganda in 2017. The extent of utilization of this model has not been fully studied. The aim of the study was to explore the patients’ and health workers’ experiences on the utilization of CCLAD model at Bwizibwera Health Centre IV, south western Uganda.

**Methods:**

This was a descriptive study employing qualitative methods. The study had 68 purposively selected participants who participated in 10 focus group discussions with HIV clients enrolled in CCLAD; 10 in-depth interviews with HIV clients not enrolled in CCLAD and 6 in-depth interviews with the health workers. Key informant interviews were held with the 2 focal persons for DSD. The discussions and interviews were audio recorded, transcribed verbatim and then translated. Both deductive and inductive approaches were employed to analyse the data using in NVivo software.

**Results:**

Patients’ and health workers’ experiences in this study were categorized as drivers and barriers to the utilization of the CCLAD model. The main drivers for utilization of this model at different levels were: individual (reduced costs, living positively with HIV, improved patient self-management), community (peer support and contextual factors) and health system (reduced patient congestion at the health centre, caring health workers as well as CCLAD sensitization by health workers). However, significant barriers to the utilization of this community-based model were: individual (personal values and preferences, lack of commitment of CCLAD group members), community (stigma, gender bias) and health system (frequent drug stockouts, certain implementation challenges, fluctuating implementing partner priorities, shortage of trained health workers and insufficient health education by health workers).

**Conclusion:**

Based on our findings the CCLAD model is meeting the objectives set out by Differentiated Service Delivery for HIV care and treatment. Notwithstanding the benefits, challenges remain which call on the Ministry of Health and other implementing partners to address these hindrances to facilitate the scalability, sustainability and the realisation of the full-range of benefits that the model presents.

**Supplementary Information:**

The online version contains supplementary material available at 10.1186/s12913-021-07105-9.

## Introduction

The burden of HIV continues to grow and as of 2020, there were over 38 million People Living with HIV (PLHIV) globally, of which Eastern and Southern Africa continues to suffer the greatest burden with close to 21 million PLHIV [1]. As of December 2019, there were 1,460,000 PLHIV in Uganda accounting for close to 3% of the national population of which 85% were on antiretroviral treatment [1]. HIV-infected populations in Sub-Saharan Africa, where the great majority of all HIV-infected persons live, are projected to survive long beyond the age of 50 as antiretroviral therapy (ART) coverage increases, resulting in an aging of the HIV pandemic in the region [2]. This, in turn, necessitates an increase in the number of qualified health professionals in an already overburdened health workforce in Sub-Saharan Africa [3, 4]. A recent study in Uganda found that, despite the reduced cost of medications, increased support from the Global Fund and the President’s Emergency Plan for AIDS Relief (PEPFAR), access to ART remains a challenge. Transportation, mobility, waiting hours at the clinic, health care personnel’s disrespect, and a shortage of ART were all mentioned as impediments to ART access [5].

In line with the aforementioned challenges and following the recommendation by the World Health Organisation (WHO) to handle the increasing number of patients in care [6], Uganda’s Consolidated HIV prevention, care and treatment guidelines, in 2016, adopted Differentiated Service Delivery (DSD) models for both HIV testing services and treatment to cater for needs and preferences of the increasing number of clients as well as improve quality of services and efficiency in service delivery [7]. The DSD refers to a strategic mix of approaches to address the specific requirements of a subgroup of clients living with HIV and in Uganda, two differentiated models were adopted; Differentiated HIV testing services and Differentiated HIV care and treatment services which aim at improved coverage, access, and quality of care. These models were based on two core principles which were client-centred care (the needs of an individual or a group of individuals) for example, HIV testing services for priority populations, provision of antiretrovirals [ARVs] at community level for stable patients and having a one stop care point for TB/HIV co-infected patients) and improved health system efficiency whilst meeting the needs of all groups of clients [8]. ﻿The recommended differentiated HIV care and treatment models for PLHIV include; Facility based models and Community-based models. The facility-based models include the Facility Based Individual Management, Fast track Drug refills and the facility-based groups whereas the Community based models include the Community Client-Led ART delivery (CCLAD) and the Community Drug Distribution Points (CDDP). In 2019, of the 1824 facilities offering ART in Uganda, 654 (36%) were reported to offer one of the DSD models [9]. Currently, the Regional Health Integration to Enhance Services in South Western Uganda (RHITES-SW) led by the Elizabeth Glaser Paediatric AIDS Foundation (EGPAF) is the implementing partner supporting the Uganda Ministry of Health on the implementation of DSD in south-western Uganda [10] .

Previous studies have noted the impact of implementing DSD models as part of national HIV programs [11] but little has been done on the follow up of implementation including the factors that may be attached to their utilization by patients and health worker satisfaction of these models in Uganda. Notably, most of these studies focused on DSD implementation as a whole and limited attention was given to individual models especially the Community Client-Led ART Delivery despite its importance in decentralizing care to community level [12, 13]. CCLAD involves a group of 3 to 12 HIV clients brought together by an expert client in the community. These clients take turns at picking medications from the health facility, while simultaneously getting assessed for viral load and other comorbidities twice annually at the health facility [14]. Importantly, previous studies have found that the Community ART delivery systems are just as efficient [15] or in certain circumstances even better than [16] facility-based models at achieving viral suppression and patient retention [17], both direct indicators of ART adherence providing re-assurance that stable patients can be safely shifted to less intensive follow up without compromising their clinical outcomes and making life long disease management more efficient for patients whilst freeing up valuable clinician time for patients requiring more intense follow-up or those being newly initiated [18].

However, it must be noted that most of these studies assessing the effectiveness of these community ART delivery models have been from controlled research settings comparing them to the traditional facility-based models [15] [16]. Additionally, other similar studies [12, 13] have been done at higher level health facilities and in urban areas where it has been noted that people in urban areas tend to prefer the facility-based models. Aforementioned, these models were meant to address the diversity of needs of people in care by being more patient-centred; increasing accessibility by reducing on transport costs and also reducing congestion at the health facilities and the workload of health workers [6]. However, in spite of all these stipulated benefits, as of 2020, the uptake of community-based models in Uganda still stood at 10% nation-wide (6% for the CCLAD and 4% for the CDDP) [19] which is low in comparison to facility-based models. Therefore, exploring factors associated with the utilization of some of these models at lower-level health facilities such as Health Centre IVs where these models are still being adopted may provide unique insights for scale up.

This study therefore sought to use detailed qualitative insights in understanding the factors related to the utilization of these community-based models in rural communities, which may have a lesser workforce and more financial need. The study was focused on exploring the supply side (healthcare system) as well as the demand side (patients enrolled and not enrolled in the CCLAD, despite eligibility). Exploring the drivers and barriers to utilization would help in understanding more about the scalability and sustainability of this model especially in rural low-resource settings. This will then inform the Government and implementing partners the extent to which CCLAD has been successfully implemented. Specifically, whether the model has been fully rolled out to accrue the expected benefits and if not, where the challenges are and what needs to be done differently to address these challenges.

## Methods

### Study design

The study followed a descriptive approach through employing qualitative methods. The study focused on exploring the barriers and drivers towards utilization of the CCLADs. It employed a case study design as it is best suited for investigating real life complex phenomena and its extensiveness [20].

The study was done in April 2021 and included Focus Group Discussions (FGDs), in-depth interviews (IDIs) and key informant interviews (KIIs). It was broadly guided by a framework of access to health care developed by Levesque et al. [21]. The framework stipulates those determinants to access to health care at various levels: providers, health facilities, health systems, (supply side), and individuals, households (demand side) and context. The Levesque framework was recently used by researchers [13] in a study of barriers to Differentiated Service ART delivery.

### Study setting

The study was carried out in the rural communities particularly targeting HIV clients registered at Bwizibwera Health Centre IV in Rwanyamahembe Sub- County, Kashari County in Mbarara district. The health centre is located 27 km by road, northwest of Mbarara city, the largest town in Western Uganda. In Uganda, a Health Center IV serves as the first referral facility providing health services in Health Sub-Districts (HSDs) where there is no Hospital and on average HSDs cover a population of 100,000 people [22]. Bwizibwera Health Centre IV was purposively selected because it has approximately 200 patients enrolled in 52 CCLAD groups, the largest number in the South Western region of Uganda.

### Study population

The study population consisted of stable HIV clients receiving HIV care and treatment at Bwizibwera Health Centre IV in South Western Uganda. Stable HIV clients are those who have been receiving ART for at least 1 year with no adverse drug reactions requiring regular monitoring, no current illnesses or pregnancy, a good understanding of lifelong adherence, and evidence of treatment success [23]. Only clients above 18 years were considered given that infants, children and adolescents are not eligible to receive services in the community [14]. Other study participants included health workers (pharmacists in charge of dispensing the medications, nurses, peer educators and lab technicians) directly involved in HIV care and treatment. The focal person for DSD at the health centre and the district focal person for HIV were also included in the study as key Informants.

### Sample selection and data collection

Using the Levesque et al. framework, different stakeholders and interview methods were purposively selected for the study. To understand the drivers and barriers towards access of HIV Care, FGDs were employed for the clients that were already enrolled in the CCLAD groups. The FGDs have the ability to generate information on how knowledge and ideas develop in a given context [24]. They also give opportunity to probe for clarification and solicit for more detailed responses [25]. These FGDs were structured based on the CCLAD groups which made mobilisation easier and participants were more open to express their different concerns around familiar groupmates. In-depth interviews were held with stable HIV clients receiving HIV care and treatment at the health facility that were not enrolled in CCLAD despite being eligible. Interviews were chosen to maintain the privacy of the HIV clients as it was noted that one of the reason these clients were not enrolled in any of the CCLAD groups was because they preferred not to expose their HIV status. In-depth interviews were also held with health workers providing HIV treatment and care at Bwizibwera Health Centre IV. Key informant interviews were held with the focal person for DSD at the Bwizibwera Health Centre IV and the Mbarara District Focal Person for HIV/AIDS. The focal persons for DSD are the overall supervisors of Differentiated Service Delivery at the health center and at district level respectively. These were specifically chosen because of their unique insights and first-hand knowledge on HIV and the implementation of Differentiated Service Delivery models. In-depth and Key Informant interviews can generate individual perspectives and experiences on the subject of interest from participants. All interviews and discussions were moderated by experienced research assistants (Bachelor degree holders) with over 5 years’ experience in research. All research assistants were taken through a training including the background and significance of the study and guidance on the study methods and tools.

The FGDs consisted of 4–6 HIV clients enrolled in CCLAD groups and were structured according to pre-existing CCLAD groups, with each FGD consisting of members of the same CCLAD group. The guide for the FGDs was adopted from a previous study [13] and was further modified to suit the study setting while the interview guides were developed by the authors (Additional file 1). Questions included in the FGD guide included what benefits and challenges the participants have identified since they joined the CCLAD, their perceived barriers to enrolment of other HIV clients in the community and to what extent the CCLAD model reflected their personal choices and decisions. The CCLAD groups were purposively selected and the peer educator at the health centre used to contact the different leaders for a date and time for the discussion to be set. Focus Group Discussions were held with the different groups until information saturation was reached after ten CCLAD groups. Each FGD lasted 45 to 60 min and the questions were structured around the drivers and barriers these clients have found with being enrolled in CCLAD groups. The FGDs were held in the homestead of the group leader or that of any of the other group members. Written informed consent was obtained from each participant before the beginning of the discussion. Each participant was given an identifying number and the discussion was audio recorded with one of the research assistants simultaneously taking notes. Stable clients that were not enrolled in any CCLAD group were purposively selected at the health centre during the ART clinic days and the in-depth interviews were held in an isolated room at the ART clinic in the health centre or at any other point of convenience for the participant. The guide for the in-depth interviews was developed by the research team, each interview lasted between 30 and 45 min and was audio recorded. Areas of focus with these clients was their knowledge on the existence of the CCLADs in the communities, why they are not enrolled and what could be done to encourage other PLHIV to join the CCLAD. Informed consent was obtained before the interview and all COVID 19 Standard Operating Procedures (SOPs) such as wearing of face masks, appropriate use of hand sanitizers and social distancing were observed.

Six in-depth interviews were also held with the health workers of Bwizibwera Health Centre IV that are directly involved in HIV care and treatment. These interviews were also held in a private room selected by the health worker at the ART clinic of the health centre after permission was gotten from the in-charge of the Health Centre. The guide for the in-depth interviews with the health workers was developed by the research team and each interview lasted between 30 and 45 min. Informed consent was obtained before the interview and all COVID 19 Standard Operating Procedures (SOPs) were observed. The area of focus for these interviews was the different problems PLHIV are facing in accessing ART in the rural communities of Bwizibwera, the effect CCLAD has had on mitigating these problems and identification of the drivers and barriers to HIV client enrolment in the CCLAD groups. Two key informant interviews were conducted with the Mbarara District focal person for HIV/AIDS and the focal Person for DSD at Bwizibwera Health Centre who were purposively selected because of their deeper and more comprehensive understanding of the CCLAD model and their positions as key person in the HIV/AIDS Control program structure. The interviews were done at their respective offices and each of them provided informed consent before the interview. COVID-19 SOPs were observed during all the discussions and interviews.

Validity was maximised by use of a uniform study guide for all discussions and all interviews, this ensured that all study participants were exposed to the same research questions which lay ground for development of the themes. For each group that is, FGDs and in-depth interviews, interviewing was done until information saturation was reached fully accounting for the sample sizes used in each case. For the health workers, all health workers directly involved in HIV care and treatment at the health facility were interviewed.

### Data management and analysis

All interviews and discussions were audio recorded and notes taken. Each recording was then transcribed verbatim and then translated into English by an independent research assistant. Each of the researchers (TKM, AM, TK, PN, JK) then familiarized themselves with all the data through independent reading of each transcript along with the field notes. The researchers then met and together inductively generated codes from the transcripts. These codes were imported into NVivo (QSR International) software which was used to generate a codebook. The codes were abstracted into subthemes which were fitted into the main thematic categories; drivers and barriers (Additional file 2). The analysis followed a framework developed by Levesque that is individual, community and health system [20]. Hence the study team adopted both deductive and inductive approaches. All research team members were fully involved in this process.

## Results

### Respondents’ characteristics

Table 1 presents the profile of the study participants by data collection method used. For the HIV clients the FDGs covered 50 participants of whom 35 were female while for the 11 IDI HIV client participants 6 were males. Furthermore, majority of the participants had only obtained primary education, had a religious affiliation and interviewees were mostly subsistence farmers.

**Table 1:** Profile of the study participants

Data collection/characteristic		NUMBER
**Key Informant Interviews participants**		**2**
*Position*		
District HIV Focal Person		1
Zonal DSD Coordinator		1
**In-depth Interviews with health workers**		**6**
*Position*		
Nurse in-charge		1
Peer counsellor		1
Counsellor		1
Clinical Officer ART Clinic in-charge		1
Lab Technician		1
Pharmacist		1
**Focus Group Discussion & In-depth Interviews with HIV Clients**		
	**FDGs**	**In-depth interviews**
*Characteristic*	*Number (n = 50)*	*Number (n = 11)*
**Gender**		
Male	15	6
Female	35	5
**Marital status**		
Married	21	10
Not married	29	1
**Highest education level**		
None	6	1
Primary	35	7
Secondary	5	2
College/University	0	1
**Religious affiliation**		
Catholic	17	5
Protestant	25	5
Pentecostal	5	1
Traditional	1	0
Muslim	2	0
**Occupation**		
Self employed	3	0
Subsistence farmer	43	5
None	1	1
Other	3	5

A filled COREQ CHECKLIST is attached as Additional file 3.

**Fig. 1:**
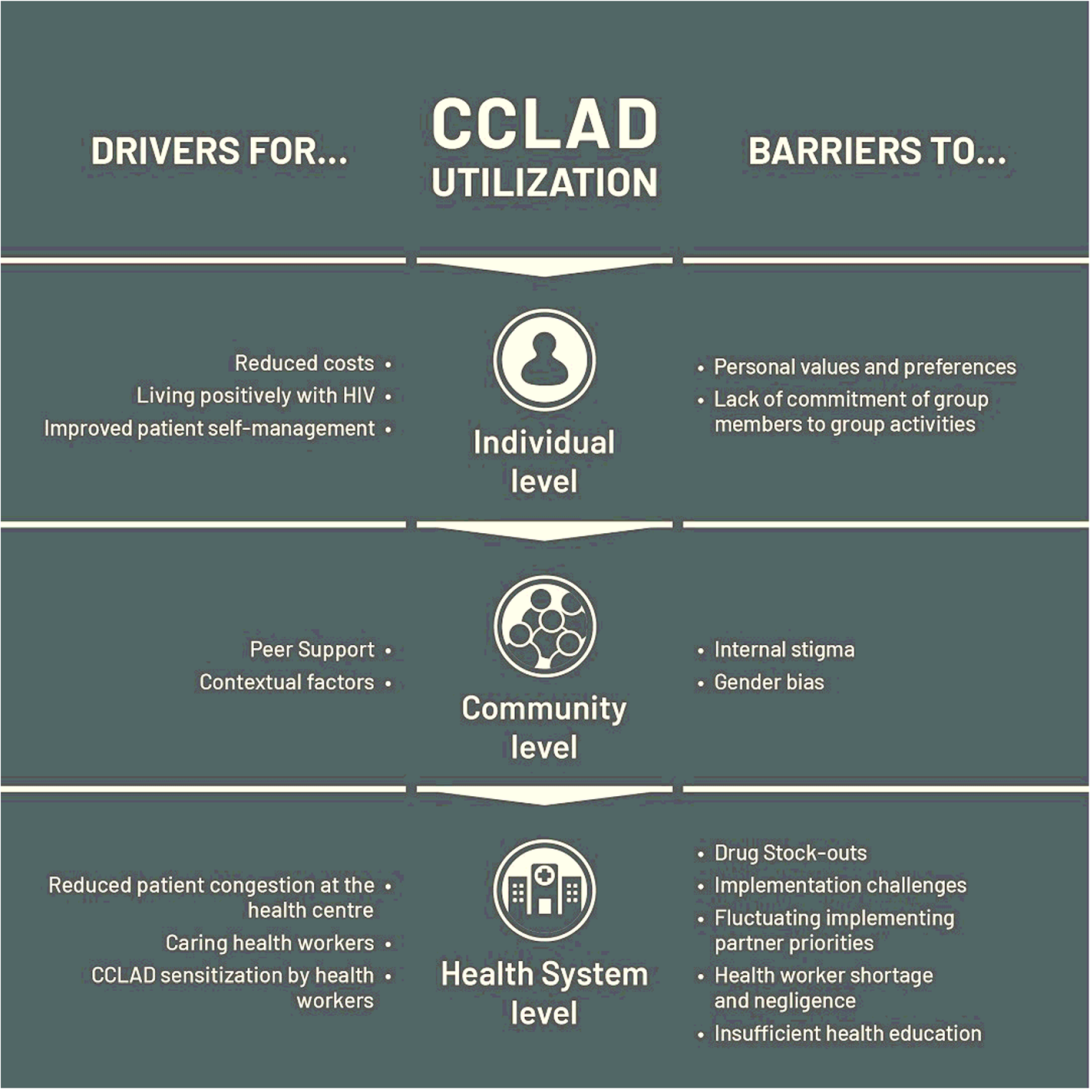
Drivers and Barriers to CCLAD utilization at Individual, Community and Health System levels

The CCLAD model was put in place to improve patient accessibility to ART while reducing the workload on the health system especially in low-resource settings. For the exploration of both the drivers and challenges to the utilization of the CCLAD model, the research team adopted an analytical framework developed by Levesque 2013, which conceptualizes the access to a health service to not only consider the supply side features of health systems but also the demand side features of the populations that is, the HIV clients. Thus, the drivers and barriers to utilization of CCLAD are structured at individual, community and health system level (Fig. 1). Verbatim quotes from the study participants are labelled in terms of the interview or discussion group number as well as the month and year in which the interview was done.

#### Drivers to the utilization of CCLAD

This study defines drivers as benefits that are being experienced by those in the CCLAD groups such that they may directly lead to increased utilization of the model or factors that have made the implementation of the CCLAD model easier when explored at individual, community, and health facility level.

##### Individual level drivers to the utilization of CCLAD

During the discussions with the patients enrolled in the CCLAD groups, three drivers to utilization at individual level were identified. These included reduced costs, living positively with HIV and improved patient self management.

### Reduced costs

This was highlighted as the most important benefit being experienced by those currently enrolled in the CCLAD groups. The groups had greatly reduced the amount of money they spent on transport to go to the health facility as they moved away from the multi-dispensing method that required them to visit the health facility once in 2 or 3 months to visiting almost once in a year; and this was only for clinical monitoring of the disease or when it was their turn to pick the medication.

*“For me number five, the good thing I have found is like I said when we had not formed a group, it was difficult to collect money to go get drugs or seeing a doctor and you would hire a motorcycle when you are one person but now it has become easy, you can call a person and you hire a motorcycle together and going to pick medicine you pay less money because only one person goes. For me that’s the good thing I have seen.” FGD-01, 04/2021*

This had also given these patients an opportunity to focus more on income-generating activities as one client said:

*“ … the benefit I have found in it is that they have made it easy for us especially the long distance of every two months. Now you can even finish eight months because one person goes, then the other one even me I stay home and do some work or sometimes I dig in my garden. That’s what I have seen.” FGD-08, 08/2021*

### Living positively with HIV

Amongst the clients in CCLAD groups, it was pointed out that they now have an increased self-esteem. Other patients reported a reduction in internalized stigma owing to the realisation that they were not in the struggle against HIV alone. These patients are now more open to disclosure of their status to other people and even offer advice to other members that may not be enrolled in the CCLAD.

*“You finish a full year without going to the hospital, you hear people saying that what happened to this person, did she get healed from HIV virus. You find people wondering about your situation and they cannot ask you directly, when are moving you here that you got healed from HIV virus. You see that, that group thing has worked for us.” FGD-03, 04/2021*

*“ … we help each other in one way or the other, we are not worried about anything even for us in our village people know us we don’t hide our HIV status from other people.” FGD-04, 04/2021*

### Improved patient self-management

Some health workers pointed out that groups have enabled patients to keep their appointments better because now, they have reduced visits to the health centre, therefore, the one or two times they get to visit the centre during the year, they make sure to see the doctor and openly share any challenges they are facing.

*“I have seen some good advantages; one patient makes up their appointments because you find that client A this time will not come, and it is their colleagues who will come … you find that a client will come to the facility once in a year and the day he comes is when he does the viral load and then goes back for another year because these other times their colleagues always come so that is one. So, the clients don’t miss out the appointments that means they won’t miss out on dozes and because they don’t miss out on their dozes that means they will keep a suppressed viral load.” HW IDI-06, 04/2021*

#### Community level drivers to the utilization of CCLAD

At community level, two main drivers emerged especially during the discussions with patients that were enrolled in the CCLAD. These were peer support and others could be broadly described as contextual factors that were favouring the formation of CCLADs in the communities.

### Peer support

Being in a group necessitates that each of the group members know about each other’s condition and as such these members are in position to offer psychosocial support to each other, a benefit that was not seen when patients were still enrolled in the facility-based models. These clients were in position to lend a helping hand when any of the other group members was facing a challenge. Additionally, through the training and benefits experienced by these clients in the groups, some of these patients have offered support to other clients in their respective communities that are not enrolled in the CCLADs. This support was in form of encouragement for these patients to come up and form CCLAD groups by preaching the numerous advantages attached to being enrolled in the CCLAD.

*“ … if one of our members gets sick and is taken to the health centre, he/she might fail to speak for him/herself so one member has to go with that sick person to the hospital so that when she/he reaches the hospital, they might not leave him/her un attended because of the sickness he/she is having concerning this group. If you are there, you are supposed to look for people to attend to him/her like 16 counsellors. They treat that person knowing what he/she is so that they can help him/her. For us that helps us as a group.” FGD-03, 04/2021*

*“But when we meet those people, we give them 17 counsellors like how our doctors taught us. We want them also to organize themselves and make a group. You find them asking you personally and you advise them what they are supposed to do. But we continue to receive them and advise them.” FGD-03, 04/2021*

### Contextual factors

Certain contextual factors were noted to ease the formation of the CCLADs, thereby increasing the utilization of the CCLAD as a model. Furthermore, this made it easier for group members to distribute drug refills and facilitated the provision of psychosocial support hence facilitating the utilization of the CCLAD. These factors could therefore be exploited to motivate the formation of groups in other areas. Most of the groups at Bwizibwera had come up because of the nearness to each other and were therefore more willing to open up to their neighbours.

*“It is not that we selected each other but as we would be at the facility you would see people from your area, we loved the idea that was brought to us. Now we said that since we are coming from the same area why not group ourselves and be like others and we went with that.” FGD-06, 04/2021*

In other cases, CCLAD groups sprouted from other groups such as financial saving groups that are already existing in the community.

*“We knew each other, so when we came, we took the responsibility to tell everyone after that we formed a group. We were 22, so every 24*^*th*^ *of every month, we would come plan and contribute some money. Then every 27*^*th*^*, we sit and receive the money we had lent to some people with interest and also lend that money to other people, at the end of the year, we share our money.” FGD-07, 04/2021*

#### Health system drivers to the utilization of CCLAD

Reduced congestion at the health facility, Caring health workers and CCLAD sensitisation done by certain health workers were among the health system drivers mostly brought out by the health workers and the patients that are enrolled in the CCLAD.

### Reduced patient congestion at the health Centre

Given that this model allows one client to represent many, there is a decrease in the number of patients at the health facility at any point in time. This is important as it decreases the workload of the healthcare providers. This allows them to focus on patients with more pressing issues. The reduced workload therefore motivates health workers to sensitize and recruit more patients into CCLAD groups thereby increasing the utilization of the CCLAD model.

*“Umm number one is that it reduces on the workload and congestion at the facility like if you are to see 80 people a day you find because of the groups they are few. So, it makes your work easier.” HW IDI-04, 04/2021*

This also leads to reduced delays in waiting time at the health facility which is associated with better quality of care and higher patient satisfaction.

*“ … there is no hustling or taking long; when you reach there and you are in a group, they welcome you, give you services and go even if you are to see the doctor and you are in a group you are the first to see the doctor.” FDG-04, 04/2021*

It also offers opportunity for quicker service delivery as most of these patients can now be seen in their respective groups as opposed to individually. This motivates patients to continuously utilize the CCLAD model.

*“When it reaches the day to see a doctor, all of us go as a group and when you reach at the doctor, it becomes easy because they receive you all at once. You go there, they direct you and you can leave fast. So, for us as a group we saw that the groups have benefits of making service delivery easy for us.” FDG-03, 04/2021*

### Caring health workers

Patients enrolled in the different CCLAD groups were appreciative of the health workers as they are passionate, caring and take their time to explain to these patients how the groups operate. In times when patients in these groups may be stigmatized, they can freely call the counsellor. Such factors are bound to improve the sustainability of these groups in the long run.

*“The health workers really treat us well; when you forget the counsellor reminds you and when you go to the facility the counsellor asks you to join the line and get treatment.” FGD-04, 04/2021*

### CCLAD sensitisation by health workers

Health workers continue to sensitize all stable patients on the existence of the CCLAD and then encourage them to go into the community to recruit other members. This continues to work well to date and is one of the factors that has favoured the rapid growth of the CCLADs at Bwizibwera Health Centre 4.

*“When I went to the hospital, I found a doctor, he explained to me about the group. Also, the counsellors told us how the groups are good and after getting the information, I also said why do not I go and gather my colleagues and form a group. So, we identified ourselves and reached a number of 6 and we went to the hospital.” FGD-04, 04/2021*

#### Barriers to the utilization of CCLAD

A number of barriers were identified at different levels – individual, community and health system. These barriers are discussed separately at these different levels in the subsequent sub-sections and could be considered as challenges the CCLAD group members are facing as well as hindrances that are making implementation of the CCLAD model difficult.

##### Individual level barriers to the utilization of CCLAD

During the interviews and discussions with the HIV clients and health workers, a number of points emerged that impede or challenge the utilization of the CCLAD model at individual level. These included one’s personal values and preferences towards management of their condition as well as lack of commitment by some group members to group activities.

### Personal values and preferences of clients

During the interviews with patients that are not enrolled in the CCLAD groups, clients were noticed to have varying needs and some of them expressed the fact that they were uncomfortable with placing the responsibility of picking the drugs into someone else’s hands.

*“Me I always want to go there myself and pick the drugs myself, tomorrow if there is anything that happens to me, I know it’s me rather than give someone my book to someone, what if they bring the wrong one or get through a wrong channel, me those are the things I fear,” IDI-10, 04/2021*

*“I know my dates I am supposed to come to the hospital, speak to my doctor and tell him. Another thing let me send someone from the group, does he/she know how I am? Hmm for me that I do not know, they might still be there but for me I am not in them. For me I come for myself,” IDI-02, 04/2021*

### Lack of commitment of group members to group activities

Another challenge that came out during the focus group discussions with the group members was the lack of commitment by some of their fellow group members. This lack of commitment manifested in many ways including failure by some group members to pick medications on time, dishonesty, and lack of integrity by some members and failure of certain group members to make timely contributions towards transport.

*“Sometimes we organize for someone to go but when the time reaches the person fails to come, if I look for that person and fail, I put in my own money and go to collect drugs,” FGD-07, 04/2021*

*“Another thing I have seen, there are people who made it their business, they pick drugs for six months but when you go to collect it, they divide it and say they were given drugs for three months so that they can get double transport when you go collect the second batch,” FGD-07, 04/2021*

*“ … the problem I see is that sometimes you go to the treasurer and you find the money is not enough there now you find you fail to go because someone has not brought the money,” FGD-01, 04/2021*

#### Community level barriers to the utilization of CCLAD

At community level, stigma was a recurring issue that came up from both the HIV clients and health workers during the interviews and discussion. Gender bias was another recurring theme especially amongst community members that had failed to join CCLAD groups.

### Internal stigma amongst the HIV clients

Internalized stigma was presented by members both in the CCLADs and those using the facility-based models as a critical barrier to the use of this model. The stigma affected patient’s enrolment in the CCLAD. One client when asked why other stable patients from the same community had not joined any group yet mentioned that:

“*Many refused just, they don’t want to be known, others have self-fear like how we normally come and meet, everyone knows the program if anyone sees you, they know that you have HIV virus. So, they do not want to be known that they have HIV/AIDs,” FGD-01, 04/2021*

Another client that had failed to join a CCLAD group despite being stable and willing said that he had failed to put together people in a group because most of them would prefer to keep their status to themselves because of the fear of stigma.

*“They don’t want people to know them, when you find her/him here, and you talk to her, they will disappear, you will never see them because the dates are not the same. They neglect you, so for me I decided let me leave the group and stay alone,” IDI-05, 04/2021*

Health workers also emphasized the fact that there was still stigma in the communities and that patients preferred to come alone to the health facility since joining a group meant that you had to disclose your status to other people.

*“Some still have the stigma; they don’t want to be known because if they join groups, it will be for the whole village and you find s/he doesn’t want his/her status to be disclosed but when s/he is alone getting his/her treatment privately and it is her/his appointment she/he goes and gets treatment and goes back home,” HW-04, 04/2021*

### Gender bias

Some clients in the communities mentioned that they had failed to join CCLAD groups because of their gender. In some cases, these were noted to be stemming from certain cultural beliefs at community level about the opposite gender for example; certain genders were rejected because of fear of breach of the group’s confidentiality whereas others were rejected without any reason given.

*“..we just loved to be only women we never wanted the problems of those men who take alcohol and when he gets drunk then he discloses your status to everyone,” FDG-09, 04/2021*

*“I found when they had formed their group, when I tried to join, those women refused, I wanted to know them. They hid from me, so I stayed alone. That is how I come alone and get my drugs,” IDI-03, 04/2021*

#### Health system barriers to the utilization of CCLAD

Some of these health system barriers were faced by the individuals who were already enrolled in the CCLADs whereas others were postulated by the health workers to be hindering the formation of new groups in the communities These included: drug stockouts, implementation challenges such as the fact that group formation is patient-initiated, insufficient health education by the health workers, health worker shortage and incompetence and delays at the health facility.

### Drug stockouts

One of the benefits of enrolling in the CCLAD is that it reduces on the number of times the clients visit the facilities. However, drug stockouts at the facilities meant that the group members could only receive medication for one or 2 months which reflected into increased transport costs.

*“ … but now the challenge they found is that medicines are not there (stock outs) and they give them medicines for only one month like those not in groups so that one also is a challenge they have. For refills they also give them one month because of stock outs. So, they wonder why they formed the groups because their interests/objectives were not fulfilled.” HW IDI-02, 04/2021*

### Difficulty in mapping or identifying other HIV clients

Formation of these groups in the community is not provider but patient initiated. This was a barrier because some of the HIV clients did not know any other HIV clients from within the same locality and so could not join or form groups.

*“R: Umm … I can say it is still at a low extent yes … because it is … it is a low extent I can say. I: Reason? R: I think one of the reasons is mapping these clients but what we have been doing of recent in their education talks ahh in their education talks they usually tell them that if you know that you come from same place with someone you can come up and form a group and they usually tell them the advantages of being in a group,” HW-IDI 06, 04/2021*

### Fluctuating priorities of implementing partners

Given implementation of DSD in south western Uganda is directly supported by implementing partners as mentioned earlier, the priorities of that partner are going to be met before what is needed by the health facility thereby directly affecting the utilization of CCLAD.

*“So, we go by the demands of the Ips. Much as it is in the guidelines, but when they bring another program which requires much attention, they first withdraw attention on CCLADs and put it on something else let us say KPs then after we go back to our CCLADs.” HW-IDI 05, 04/2021*

### Health worker shortage and negligence

Given that the number of health workers who are trained in DSD remain low, they are not in position to educate the large volume of clients on what CCLAD is or how it operates. Health workers are also reluctant to switch to this mode owing to the increased amount of workload associated with it in terms of records keeping. Other health workers were noted to be negligent towards their work and preferred to go on long breaks which from the patient’s point of view undermined the very reason they had joined CCLAD groups so as to save more time and spend less time at the health facility.

*“All over the world the hardest thing is documenting; tell people to write you are causing problems now you are telling them to add on writing people into another register, assigning them another group it is an additional work, so you are increasing work at a particular time of formation. Then teaching, if someone has not grasped the concept then you will not translate the information and if the other person does not understand what it means also, they will take wrong information and you know how information and communication can cause issues so those are the challenges.” KII-01, 04/2021*

*“If you find them not in the moods of working, you get disturbed a lot, you got to the lab to pick blood samples from you and you find they are not there. I don’t know what I face if it is the same as what my friends face.” FGD-07, 04/2021*

### Insufficient health education

Some HIV clients claimed that their counterparts in the communities did not know about the existence of the groups. Additionally, from the interviews it was gathered that, some clients held misconceptions about the groups given that they had not properly been educated on how these groups operate. One client when asked about the groups had this to say:

*“I do not know them, because I come and pick my drugs and I go back home. They give me dates to come back, for me they have not taught me to join that group.” IDI-02, 04/21*

During one of the discussions, one client that had approached another HIV client was told:

*“ … in fact, there is one I approached and asked to join the group and she said that those groups are for the poor only that if I have my transport why would I join the group.” FGD-04, 04/21*

## Discussion

This study investigated the drivers and barriers to the utilization of the CCLAD by exploring the views of both the health workers and the HIV clients. Drivers were considered to be benefits that are being experienced by those in the CCLAD such that they may directly lead to enrolment of more clients and as such increased utilization e.g., reduced costs, improved peer support, living positively with HIV, improved patient self-management, reduced workload, and delays. Additionally, other factors that were making CCLAD implementation easier were also considered to be drivers and these included CCLAD sensitisation, caring health workers and context. Challenges were also explored following the same approach; those that have hindered utilization of the model such as implementation challenges, personal values and preferences, stigma and gender bias, insufficient health education, mobility of some HIV clients, insufficient health education and challenges faced by patients that are already enrolled including lack of commitment of some group members and frequent drug stockouts.

In the 2017 implementation guidelines, CCLAD was defined as a psychosocial community ART groups comprising of stable ART clients in the same community [14]. The psychosocial aspect, in form of peer support, is one that was greatly emphasized by patients in these groups. This support is unique in that it was offered by people who share common experiences or have faced similar challenges that is, by those in groups to themselves and those not in the groups. The support offered was reported to reduce internalized stigma and encourage positive living amongst patients. The support also emerged at community level as some clients were noted to have become advocates for these groups.

Additionally, the CCLAD model has proven beneficial especially in low resource settings, where the reduced volume of patients means that health workers are not strained. It also allows health workers to give patients much more attention and time leading to improved quality of care hence improved patient satisfaction even for those that are not enrolled in the groups. Less lines also means better quality of care. Additionally, health workers also reported improved patient self-management as patients became more vigilant and felt more empowered, occurrences that may translate into improved adherence and hence a greater percentage of patients with a suppressed viral load. This is key to achieving the UNAIDS 95–95-95 goal by 2030. According to the 2016 MOH consolidated guidelines, the second principle of DSD was improved health system efficiency [7] which is directly in line with the findings of this study. Therefore, the facilitators that have been identified by this study underscore most of the objectives of community-based ART delivery set out by the WHO in 2014 [6] and the Ministry of Health when it was recommending their use in 2016 [7].

Certain drivers identified in this study, such as the fact that geographical proximity made it easier to form groups could be exploited by implementing partners and other stakeholders. Therefore, by moving towards a provider-initiated approach, service providers can map stable clients that come from the same area, educating them on the benefits of this model thereby encouraging them to enrol. However, with a provider-initiated approach, there would be need for extra training for the group members since one of the pearls for sustainability under the patient-initiated groups is that members choose other members that they are comfortable with. Implementing partners should also consider existing structures in communities such as Savings and Credit Co-operatives (SACCOs) as starting points for groups or such financial groups can also be incorporated into existing CCLAD groups as they may offer more motivation and incentive to clients. Other drivers such as caring health workers should be a pointer to health workers as to what clients expect. It is important to emphasize principles such as empathy, being caring and having good communication skills as these increase the level of trust between patient and health worker, thereby making it easier for them to take up their advice. Some of our study findings are similar to those from a study conducted in Malawi to understand the challenges and successes of DSD models that identified reduced travel time, reduced visiting time, decreased congestion at health facilities and increased social support as some of the benefits of the models [26].

Despite all the above, there are still a number of barriers to the utilization of the CCLAD model. Important to note from the findings is that some of these challenges faced by members in the groups are likely to paint a negative picture of the CCLAD and thereby discourage other clients from joining them. Challenges like lack of commitment of certain members in a group are quite personal and relate to group dynamics and similar findings were highlighted in Malawi [26]. These highlight the need for continuous health education even after the patients have joined the groups and ongoing counselling so that members can be continuously motivated to keep being part of the group. There is also need for implementing partners and stakeholders to continuously invest in training health workers. The extent of utilization of this model mostly lies on the shoulders of trained health workers as they are the ones to guide recruitment and training of prospective group members and offer the continuous support. Therefore, there can be no health education if the health workers themselves are not adequately trained.

As evidenced by the findings of drug stockouts being a barrier, there is a need to maintain frequent drug supply to uphold the efficiency principle of the model. The lack of medication forces the health workers to only offer supplies for one or 2 months which directly undermines the move away from multi-drug dispensing and the savings on transport costs which are the main stand-out features of this model. In the 2014 WHO supplementary guidelines, it was pointed out that the success of community ART models depends on sufficient and reliable support and resources, including a flexible and reliable drug supply, access to quality clinical management, a reliable monitoring system that can follow patients in and out of the community to the clinic and a nationally supported cadre of lay health workers. A number of the barriers that have been highlighted were previously explored in more urban and semi-urban settings in Uganda [12, 13] and seem to recur even in rural settings, thereby highlighting the reliability of our findings.

Considering, this study was done in a rural setting, most of the findings are not generalisable and therefore it is important that the views of HIV clients from more diverse settings are focused are studied. Because of the qualitative nature of this research, how participants “place” the researchers may have significant effects on the findings. Therefore, all discussions and interviews were done by research assistants who had no attachment to the health facility where the study participants get HIV care and treatment. At the beginning of each discussion, it was stressed by the research assistants that there was no correct or wrong answer, and all study participants were encouraged to express their feelings and thoughts freely and openly freely and openly. Validity was further maximised by use of uniform study guides for the discussions and interviews, this ensured that all study participants were exposed to the same research questions which lay ground for development of the themes. For each group that is, FGDs and in-depth interviews, interviewing was done until information saturation was reached fully accounting for the sample sizes used in each case. However, for the health workers, all health workers directly involved in HIV care and treatment at the health facility were interviewed. There is need for future studies to look at the views of larger samples of patients and compare this across rural areas as this study was based off a small proportion of patients who were all reporting to the same health facility.  However, health centres all have different management and some centres are more likely to serve patients beyond the programmed catchment area. Therefore, findings from this study may represent the views of diverse clients and so may be extrapolated to represent those of the country.

## Conclusion

This study highlights the experiences (both drivers and challenges) of patients and health workers as regards CCLAD utilization at individual, community and health system level. According to our findings, the main drivers for utilization of this model were: at individual level (reduced costs, living positively with HIV, improved patient self-management), community level (peer support and contextual factors) and health system level (reduced patient congestion at the health centre, caring health workers as well as CCLAD sensitization by health workers). These findings complement the objectives set out by the World Health Organization in 2014 when recommending decentralized models of HIV care and treatment and those set out when community-based models were being rolled out by the Ministry of Health, Uganda in 2016. However, significant barriers to the utilization of this community-based model were also identified at similar levels: individual level (personal values and preferences, lack of commitment of CCLAD group members), community level (stigma and gender bias) and at health system level (frequent drug stockouts, difficulty in mapping or identifying other HIV clients, fluctuating implementing partner priorities, shortage of trained health workers and insufficient health education by health workers). These findings highlight gaps and areas, most of which are at health system level, that need to be taken into consideration by implementing partners and the Ministry of Health so as to fully benefit from decentralization of HIV care and treatment. Lessons drawn from these findings can guide the scalability, introduction of these models of care in other low-resource settings and sustainability of these models in areas where they are already active.

## Supplementary Information

**Additional file 1.**

**Additional file 2.**

**Additional file 3.**

## Data Availability

﻿The datasets generated during and/or analyzed during the current study are not publicly available due to ethical reasons but are available from the corresponding author on reasonable request.

## Abbreviations

ART: Anti-retroviral therapy
ARV: Anti-retrovirals
CCLAD: Community Client-Led ART Delivery
CDDP: Community Drug Distribution Points
DSD: Differentiated Service Delivery
FGDs: Focus Group Discussions
HSDs: Health Sub-Distrcits
IDIs: In-depth Interviews
KIIs: Key Informant Interviews
MoH: Ministry of Health
PEPFAR: President’s Emergency Plan for AIDS Relief
PLHIV: People Living with HIV
SACCOs: Savings and Credit Co-operatives
SOPs: Standard Operating Procedures
UNAIDS: Joint United Nations Programme on HIV/AIDS
WHO: World Health Organization
