# Supplementary material for: Exploring drivers and barriers to the utilization of community client-led ART delivery model in South-Western Uganda: patients’ and health workers’ experiences
Source: BMC Health Serv Res. 2021 Oct 20;21:1129. doi: 10.1186/s12913-021-07105-9 (PMC8527820; doi:10.1186/s12913-021-07105-9)
Supplement: Supplementary file 1 — Additional file 1. [file 12913_2021_7105_MOESM1_ESM.pdf]

COMMUNITY CLIENT LED ART DELIVERY (CCLAD) UTILIZATION IN SOUTH WESTERN UGANDA – STUDY TOOLS

1. FGD TOPIC GUIDE FOR HIV CLIENTS IN CCLAD GROUPS

DISCUSSION QUESTIONS. *Participant socio-demographic will be collected through a different form.*

Introductory

1. Please briefly tell me about yourself and how long you have been on ART.
2. Before the introduction of the CCLAD, how frequently did you go to the facility for scheduled reviews (including ART refills)?
3. When and how did you come to be enrolled in the CCLAD?

Individual-level factors

1. Are you aware of the existence of DSD models?

2. Please briefly tell me about the CCLAD. How did you come to be enrolled in it?

3. What advantages do you find in the CCLAD? (*Probes: i) Savings in time ii) savings in transport costs iii) benefits of less time away from work iv) reduced congestion.)*

4. What challenges, if any, are you experiencing with these CCLAD? *(Probes: drug stock-outs; irregularities in supply; community participation, attitudes towards the healthcare workers)*

Health-system factors

5. CCLAD is meant to be *patient-centered.* What is your comment on the extent to which current CCLAD reflects your personal choices and preferences?

Community factors

6. Tell me about barriers to uptake of CCLAD? (*Probes patient literacy of DSD models, demand-creation campaigns prospects, stigma).*

7. Tell me about the enrollment and participation of other HIV clients in your locality to a CCLAD. *(Probe on refusal to enroll, the level of participation)*

8. Is there anything you would want to share with me about the CCLAD in your locality?

2. INTERVIEW GUIDE FOR HIV CLIENTS NOT UNDER CCLAD

Interview Questions:

1. Tell me about yourself and how long you have been on ART.
2. Tell me about what challenges you face as an HIV client in this community. *(Probe on accessibility of ART and how frequently they visit the health care center for refills and reviews)*
3. Tell me about any solutions that have been formulated to overcome the challenges.
4. Tell me whether you know anything about the DSD models. *(Probe on knowledge about CCLAD and what they know about them)*
5. Tell me why you have chosen not to be enrolled in the CCLAD.
6. Is there anything else you want to share with me?

3. INTERVIEW GUIDE FOR HEALTH WORKERS AND KEY INFORMANTS

Interview Questions:

1. Tell me about the challenges PLHIV in rural communities in Bwizibwera face? *(Probe on access to ART; what has been done to overcome these—DSD; CDDP)*
2. Tell me about the uptake of the CCLAD. (*Extent of uptake* & reason.) *Probe on patient and community involvement or if not, understand why?)*
3. Tell me about the benefits you have achieved from using the CCLAD. *(Probe on work load, more time to focus on more critical patients, congestion at health centers)*
4. Tell me about the challenges you have faced with the use of these models? (Probe on drug availability, evidence of stigmatization)
5. Tell me about any suggestions to address the said challenges.
6. Is there anything else you want to share with me?
