# Supplementary figures and images for: Exploring drivers and barriers to the utilization of community client-led ART delivery model in South-Western Uganda: patients’ and health workers’ experiences
Source: BMC Health Serv Res. 2021 Oct 20;21:1129. doi: 10.1186/s12913-021-07105-9 (PMC8527820; doi:10.1186/s12913-021-07105-9)

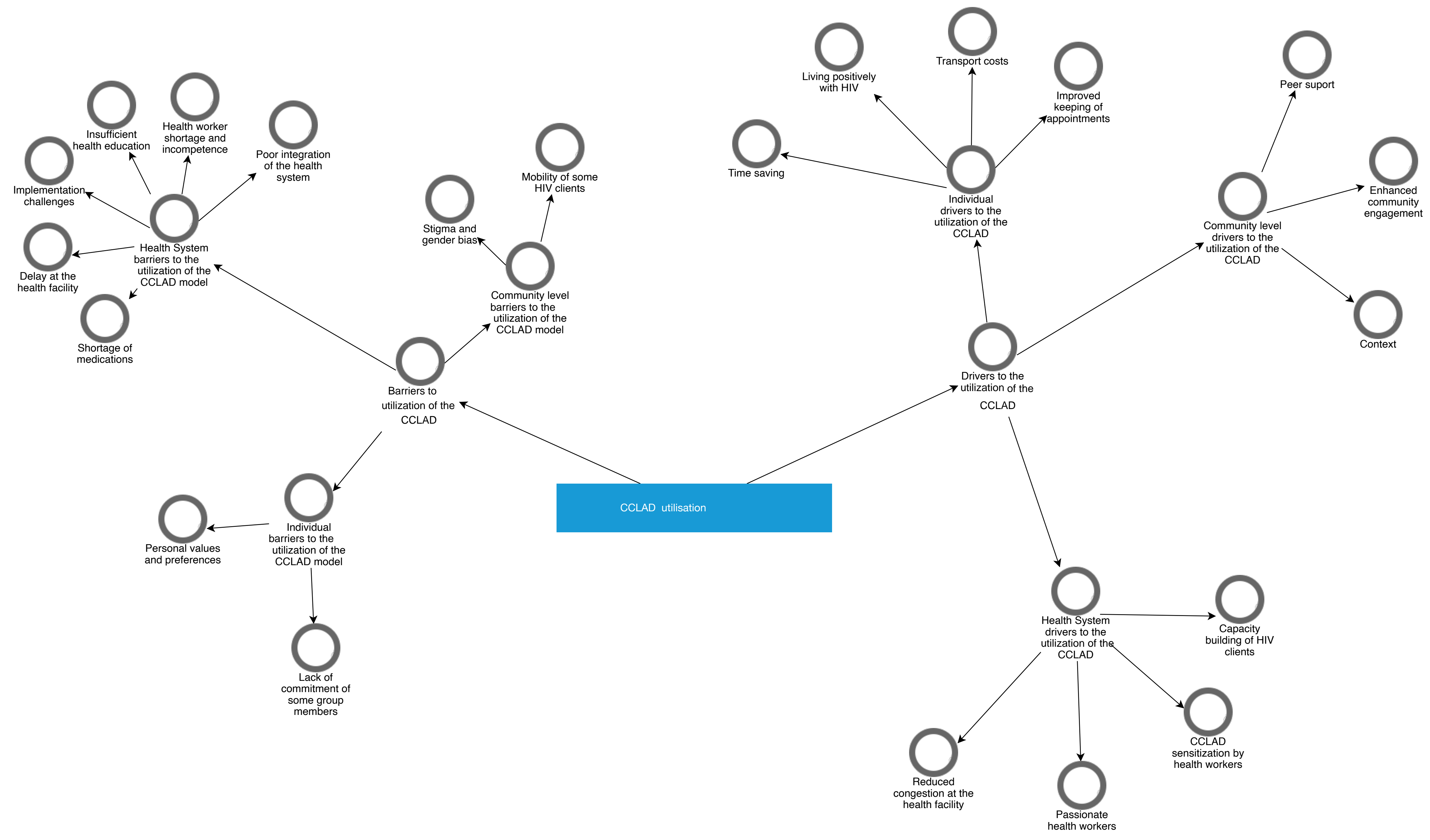

Supplement: Supplementary file 2 — Additional file 2. [file 12913_2021_7105_MOESM2_ESM.pdf]
