# Supplementary material for: Exploring drivers and barriers to the utilization of community client-led ART delivery model in South-Western Uganda: patients’ and health workers’ experiences
Source: BMC Health Serv Res. 2021 Oct 20;21:1129. doi: 10.1186/s12913-021-07105-9 (PMC8527820; doi:10.1186/s12913-021-07105-9)
Supplement: Supplementary file 3 — Additional file 3. [file 12913_2021_7105_MOESM3_ESM.pdf]

COREQ Checklist _Utilization of CCLAD in Uganda

NO. item	Description	Reported on page#
**Domain 1: Research team and reflexivity**		
Personal characteristics		
1. Interviewer/ facilitator	Interviews were carried out by trained Research Assistants- Arnold Kamugisha (AK) and Doreen Ainembabazi (DA)- under the supervision of Timothy Mwanje Kintu (TMK), Anna Maria Ssewanyana (AMS), Tonny Kyagambiddwa (TK), Pretty Mariam Nampijja (PMN) , Patience Kevin Apio (PKA), Jessica Kitaka (JK) , Jerome Kahuma Kabakyenga (JKK)	Page 5 under sample selection and data collection
2. Credentials	TMK, AMS, TK, PMN, PKA and JK are Undergraduate health professions students. JKK is a PhD holder.	N/A
3. Occupation	6 of the authors are students under the Faculty of Medicine and the corresponding author is a Professor of Public Health.	N/A
4. Gender	4 females and 3 males	N/A
5. Experience and training	HIV/AIDs health services and research through the Health Education Partnership Initiative- Transforming Ugandan Institutions Training Against HIV/AIDS (HEPI-TUITAH_ https://hepi-tuitah.must.ac.ug/). All authors were trained in the Responsible Conduct of Research prior to the study.	N/A

	Research Assistants received 3 day training on the details of the study.	
Relationship with participants		
6. Relationship established	Relationship wasn't established with participants prior to study commencement.	N/A
7. Participant knowledge of the interviewer	Participants understood that the researchers were there to collect information on their experiences with the CCLAD model so that they can improve on service delivery.	Page 5 - under sample selection and data collection
8. Interviewer characteristics	Research Assistants -AK and DA are Bachelor's degree holders with 5 years research work experience.	Page 5 - under sample selection and data collection
**Domain 2: Study design**		
Methodological framework		
9. Methodological orientation and theory	Descriptive Case study approach using Levesque et al. framework [22]	Pages 4 and 5
**Participant selection**		
10. Sampling	Participants purposively selected	Page 5 sample selection
11. Method of approach	Participants approached face-to-face in focus group discussions and in-depth interviews	page 5 – data collection
12. Sample size	68 participants	pages 8-10 sample selection & data analysis.
13. Non-participation	No one refused to participate and no one dropped out of the study	N/A
Setting		
14. Setting of data collection	Study was carried out in the rural communities and at the health centers.	pages 5-7 sample selection and data collection.

15. Presence of non-participants	No one else was present apart from the researchers and participants	pages 5-7 sample selection and data collection.
16. Description of sample	Sample included 8 health care workers and 60 HIV clients at the health facility (15 males and 35 females)	Pages 5-7 in table 1.
**Data collection**		
17. Interview guide	FGD topic guide was adopted from Zakumumpa et al. [12] and made fit for this particular study wile interview guides for IDIs and KIIs were developed by authors. Questions, prompts, and guides were provided but were not pilot tested.	page 5 -sample selection and data collection
18. Repeat interviews	Repeat interviews were not carried out	N/A
19. Audio/visual recording	The research used audio recording	Methods; page 7
20. Field notes	Field notes were made during the focus group discussions and in-depth interviews.	Methods; page7
21. Duration	The average duration of the group discussions and in-depth interviews was 1hour.	Methods; page 7
22. Data saturation	Interviews and Discussions were held until information saturation.	Methods; page7
23. Transcripts returned	Transcripts were not returned to participants for comment or correction.	N/A
**Domain 3: Analysis and findings**		
Data analysis		
24. Number of data coders	5 data coders	Under data analysis and management; page 9

25. Description of the coding tree	Two main themes; drivers and barriers were obtained. Under-each of these; sub-themes were identified at individual, health system and community levels.	Under data analysis and management; pages 9
26. Derivation of themes	Sub-themes were inductively from existing research. Major themes and the different levels derived deductively and a framework developed by Lavesque et al. [20]	Page 9 - under data analysis and management.
27. Software	NVivo (QSR International) software	page 9 - Data Analysis and Management;
28. Participant checking	Participants didn't provide feedback on the finings	N/A
Reporting		
29. Quotations presented	Verbatim quotes presented to illustrate findings. Identified as FGD and IDI numbers.	page 12-30 - results
30. Data and findings consistent	Both data and findings were consistent.	N/A
31. Clarity of major themes	Major themes were presented with accompanying explanatory texts.	pages 12-30
32. Clarity of minor themes	Explanatory texts provided for sub-themes.	Pages 12-30
